# In Situ Light-Source Delivery During 5-Aminulevulinic Acid-Guided High-Grade Glioma Resection: Spatial, Functional and Oncological Informed Surgery

**DOI:** 10.3390/biomedicines12122748

**Published:** 2024-11-30

**Authors:** José Pedro Lavrador, Francesco Marchi, Ali Elhag, Nida Kalyal, Engelbert Mthunzi, Mariam Awan, Oliver Wroe-Wright, Alba Díaz-Baamonde, Ana Mirallave-Pescador, Zita Reisz, Richard Gullan, Francesco Vergani, Keyoumars Ashkan, Ranjeev Bhangoo

**Affiliations:** 1 Department of Neurosurgery, King’s College Hospital NHS Foundation Trust, London SE5 9RS, UK; francesco.marchi@eoc.ch (F.M.); m.awan1@nhs.net (M.A.); k.ashkan@nhs.net (K.A.);; 2 Department of Neurosurgery, Neurocenter of Southern Switzerland, Ente Ospedaliero Cantonale, 6900 Lugano, Switzerland; 3 Department of Neurophysiology, King’s College Hospital NHS Foundation Trust, London SE5 9RS, UK; 4 Department of Clinical Neuropathology, King’s College Hospital NHS Foundation Trust, London SE5 9RS, UK

**Keywords:** 5-ALA, blue-light source, glioblastoma, surgery, Spectra, MIPS, Nico Myriad, Gliolan

## Abstract

Background/Objectives: 5-aminulevulinic acid (5-ALA)-guided surgery for high-grade gliomas remains a challenge in neuro-oncological surgery. Inconsistent fluorescence visualisation, subjective quantification and false negatives due to blood, haemostatic agents or optical impediments from the external light source are some of the limitations of the present technology. Methods: The preliminary results from this single-centre retrospective study are presented from the first 35 patients operated upon with the novel Nico Myriad Spectra System©. The microdebrider (Myriad) with an additional in situ light system (Spectra) can alternately provide white and blue light (405 nm) to within 15 mm of the tissue surface to enhance the morphology of the anatomical structures and the fluorescence of the pathological tissues. Results: A total of 35 patients were operated upon with this new technology. Eight patients (22.85%) underwent tubular retractor-assisted minimally invasive parafascicular surgery (tr-MIPS). The majority had high-grade gliomas (68.57%). Fluorescence was identified in 30 cases (85.71%), with residual fluorescence in 11 (36.66%). The main applications were better white–blue light alternation and visualisation during tr-MIPS, increase in the extent of resection at the border of the cavity, identification of satellite lesions in multifocal pathology, the differentiation between radionecrosis and tumour recurrence in redo surgery and the demarcation between normal ependyma versus pathological ependyma in tumours infiltrating the subventricular zone. Conclusions: This proof-of-concept study confirms that the novel in situ light-source delivery technology integrated with the usual intraoperative armamentarium provides a spatially, functionally and oncologically informed framework for glioblastoma surgery. It allows for the enhancement of the morphology of anatomical structures and the fluorescence of pathological tissues, increasing the extent of resection and, possibly, the prognosis for patients with high-grade gliomas.

## 1. Introduction

5-aminolevulinic acid (5-ALA)-guided surgical resection is a well-established technique in high-grade glioma surgery, particularly for glioblastomas. Since the initial work by Stummer et al. [1,2,3], multiple neuro-oncology groups have reported the benefits of using this surgical aid to improve extent of resection (EoR) [4,5,6,7,8,9], progression-free survival (PFS) [10,11] and overall survival (OS) [12,13]. In some countries, such as the United Kingdom, it is part of the standard of care in the surgical treatment of patients diagnosed with presumed glioblastomas (GBMs), in which Gross Total Resection is considered achievable [5,14].

The impact of this on the neuro-oncology community paved the way for intense research and development in the fields of intraoperative optics, light sourcing, luminescence and imaging [15,16,17] and the advances provided by this adjunct for patients with high-grade gliomas. Multiple intraoperative microscopes, exoscopes and endoscopes [18,19,20] and head lamps and loupes [21,22] are now equipped with a blue-light source around a 400 nm wavelength to incorporate this innovation and assist surgeons intra-operatively.

However, there remains uncertainty about the impact of the location of the light source and its distance to the surgical field on the intensity of the perceived fluorescence [23,24]. Moreover, its correlation with imaging features in both structural MRI and advanced imaging (perfusion and permeability studies and spectroscopy and PET) [25,26,27], histological tumour infiltration [28,29] and the potential for false positives [30,31] requires further clarification. This is crucial given the rapidly evolving field of the classification of EoR in GBM, particularly the concept of supramaximal or supratotal resection, [32,33] and its implications in the prognostication of the disease [34].

In this paper, we present a new technology that delivers the blue-light source responsible for 5-ALA-related fluorescence in situ—the Nico Myriad Spectra System©. We share our initial experience and the potential applications of this technique within the existing field of high-grade glioma surgery.

## 2. Materials and Methods

This is a retrospective single-centre study of patients who underwent surgery with the in situ light-source delivery of both white and blue light (405 nm) from March to September 2024 with a presumptive diagnosis of high-grade glioma after clinical and imaging discussion by a neuro-oncology multidisciplinary team [35,36,37].

This work was conducted in compliance with the current version of the Declaration of Helsinki and the ICH-GCP or ISO EN 14155 (as far as applicable), as well as all national legal and regulatory requirements and the ethical standards of our institution. The inclusion criteria consisted of patients aged > 18 years, presumed or possible differential diagnosis of high-grade glioma on the preoperative MRI, both elective and emergency surgery, preoperative administration of 5-ALA and a valid consent form for the procedure.

All surgeries were performed with the Stealth Medtronic© system (Medtronic Sofamor Danek, Memphis, TN, USA) for neuronavigation. Intraoperative neuromonitoring and mapping were used in all surgeries to assess motor (continuous direct cortical motor evoked potentials—MEPs) using a subdural strip electrode, transcranial MEPs using cork screws and continuous dynamic subcortical stimulation with the high-frequency technique [38] and, where appropriate, visual (visual evoked potential monitoring using subdural strip and cork screws) [39] and language (cortical and subcortical low-frequency technique) [40] functions in tumours within the vicinity of the relevant cortical–subcortical eloquent areas.

In all cases, 5-ALA administration was performed within 2 hours of signing in to the surgical theatre using an oral route with a dose of 20 mg/kg up to a maximum dose of 1500 mg per patient, as previously published by our group [41].

**Figure 1:**
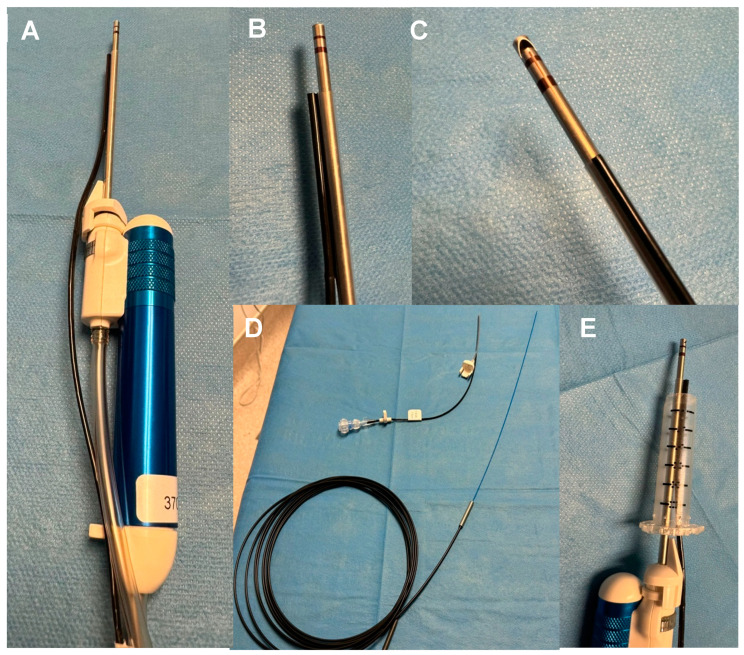
Nico Myriad Spectra System©. (**A**) The hand-held device with the full equipment assembled. (**B**) The tip of the device with the microdebrider and the light source. (**C**) The tip of the microdebrider with the lateralised cutting edge and the light source. (**D**) The long cable of the light source (at the bottom of the figure), which is inserted in the flexible black cable (above in the figure) attached to the microdebrider. (**E**) The fully assembled device inside the BrainPath tubular retractor.

**Figure 2:**
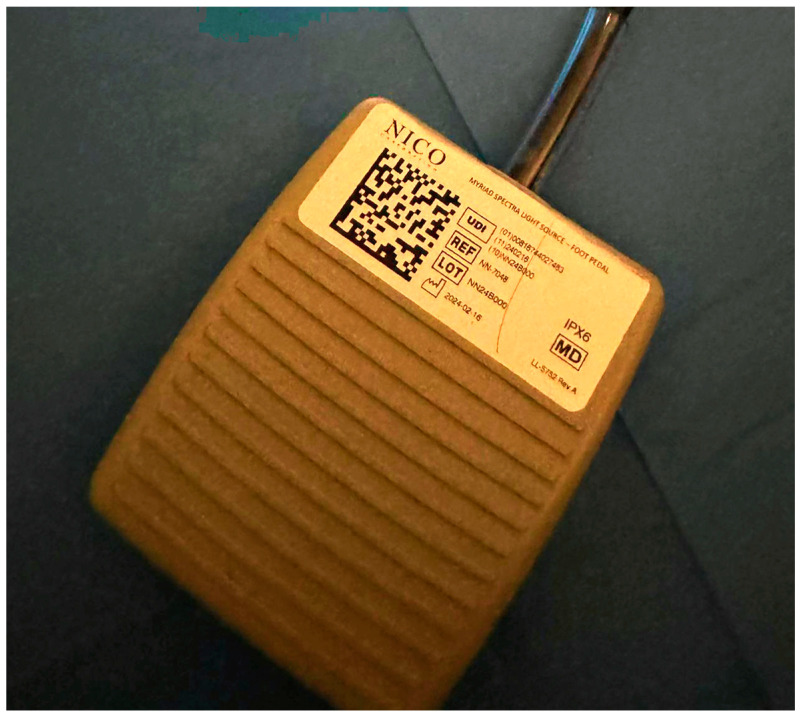
Foot pedal of the system.

**Figure 3:**
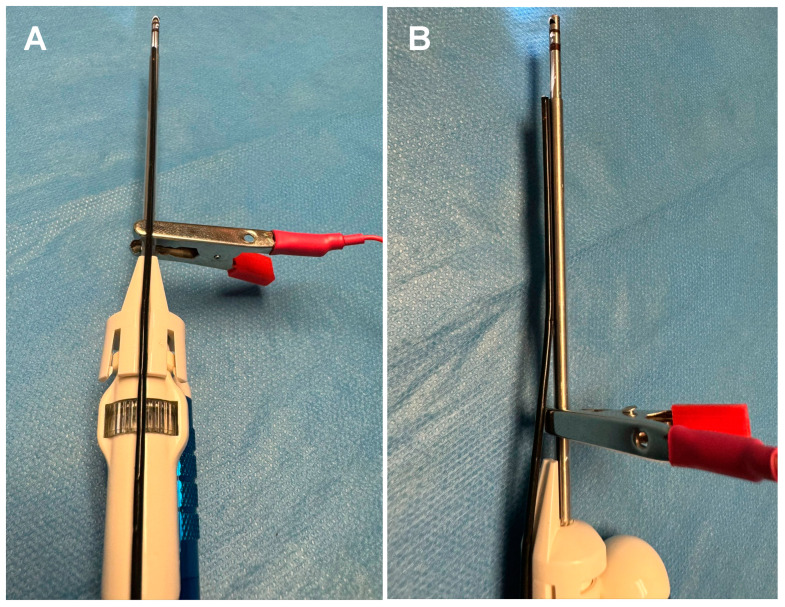
(**A**) Nico Myriad Spectra System© electrified with sterile disposable alligator clip. (**B**) Close-up view of the alligator and the device with the electrification of the microdebrider through a snip cut in the plastic sleeve of the Spectra NICO Myriad.

The Nico Spectra Light Source© is a recently FDA- and MHRA-approved specific component of the multi-functional medical device Nico Myriad Spectra System© (Nico Corporation Indianapolis, IN, USA) (Figure 1A–E). This system allows for the resection and collection of the neoplastic tissue and, thanks to the addition of the directional light source close to the surgical field, provides a better understanding of the surgical area of interest and better tumour visualisation. The Spectra hand-held device enables us to switch easily between white and blue light by pressing a foot pedal (Figure 2 and Video S1a,b). The Spectra Nico Myriad was used simultaneously with the intraoperative microscope (Kinevo 900 Zeiss©, Pentero, Carl Zeiss AG, Oberkochen, Germany). Using either the white light or the 400 nm wavelength light source from the microscope, the Spectra Nico Myriad was used with either white light or blue light (405 nm) at 100% (Video S2). The integration of IONM with the Myriad microdebrider is crucial, particularly in tubular retractor MIPS given the reduced degrees of freedom to manipulate instruments, which mandates the extraction of maximal information from a single instrument in the surgical field. Therefore, similar to a previous report from our group [42], we electrified the Myriad microdebrider through a cut in the plastic sleeve of the Spectra Nico Myriad [42] (Figure 3).

## 3. Results

Our study includes 35 patients, of whom 15 were females (42.85%) and 20 were males (57.15%), with a mean age of 52.6 ± 15.17 years old (Table 1). The histopathology demonstrated 24 as high-grade gliomas (68.57%), 4 as low-grade gliomas (11.42%), 3 metastases (8.57%), 1 lymphoma (2.85%), 1 medulloblastoma (2.85%), 1 meningioma (2.85%) and 1 subependymoma (2.85%). A total of 8 of the 35 patients (22.85%) were operated with the tubular retractor-assisted minimally invasive parafascicular surgery (tr-MIPS) approach. Gross Total Resection (GTR) was achieved in 20 cases (57.15%) whilst a Subtotal Resection (STR) was achieved in the remaining 15 cases (42.85%). None of the low-grade gliomas or lymphomas showed any fluorescence both under the blue light of the microscope and of the Spectra. The remaining 30 cases (85.71%) showed avid fluorescence. Among them, in 19 out of the 30 cases (63.33%), no fluorescence was identified at the end of the resection, while in 11 cases (36.66%), the cavity showed remnant fluorescence both with the microscope and the Spectra, but further resection was not pursued given the proximity to functional borders.

### 3.1. Main Applications

#### 3.1.1. Tubular Retractor-Minimally Invasive Parafascicular Surgery (tr-MIPS)

**Figure 4:**
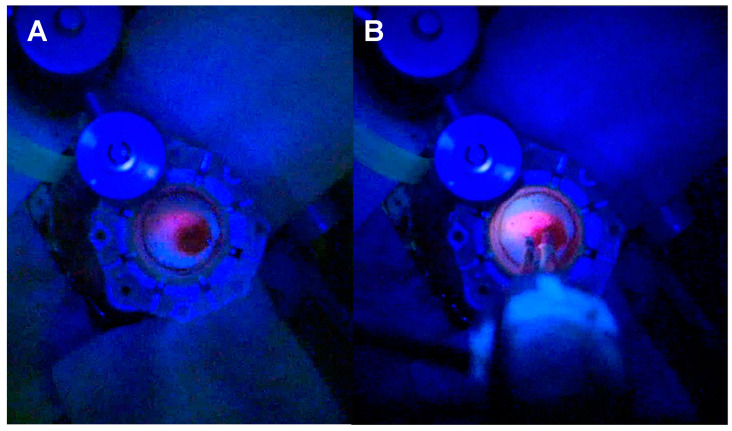
Comparison between visualisation of fluorescent tumour tissue in tr-MIPS under the blue light of the microscope without (**A**) and with (**B**) Spectra.

**Figure 5:**
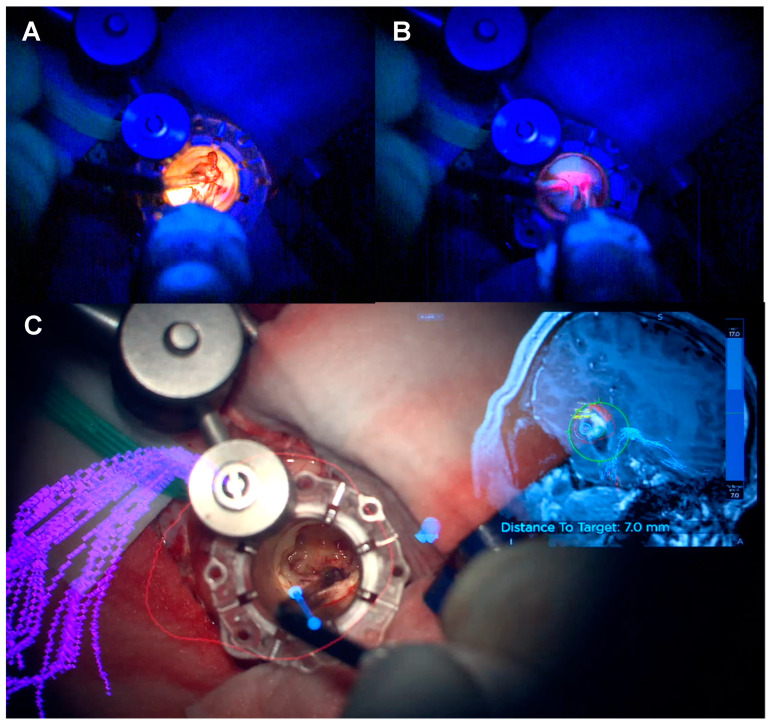
Application of Spectra within the tubular retractor in tr-MIPS. (**A**) Delivery of the white light to maximise the anatomical information. (**B**) Delivery of the blue light for the functional information. (**C**) Integration of the system with the neuronavigated microscope.

Visualisation is critical during tubular retractor-assisted MIPS given the limited degrees of freedom for bimanual dissection. Dependency from an external light source—loupes, exoscope or microscope—mandates a co-axial location in the direction of the tubular retractor to maximise light at the depth of the surgical field, although this limits the ability to work at the edges of the cavity [43,44,45]. Spectra allows for the light source to be delivered next to the tip of the instrument at use, bypassing the physical limitations and barriers of the tubular retractor light source when this is external, and maximising the manoeuvrability and visualisation during resection (Figure 4 and Video S3). In addition, it overcomes the concern about “false negatives” when the blue light is provided by an extra-tubular source, reassuring the surgeon that the blue light reaches the tissue of interest with no interposition of physical obstacles. Figure 5 and Video S3 demonstrate how the delivery of the light source within the tubular retractor maximises the anatomical information obtained with the white light and the functional information with the blue light.

#### 3.1.2. Extent of Resection (EoR) in Patients Eligible for Gross Total Resection

**Figure 6:**
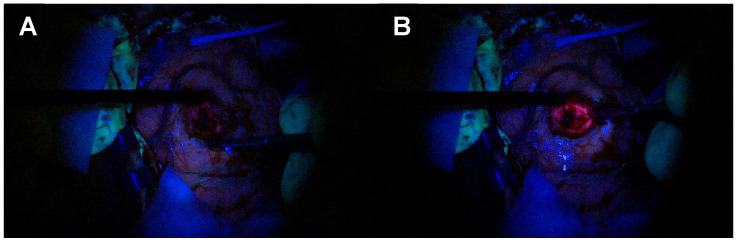
EoR in patients eligible for Gross Total Resection. Comparison between residual fluorescence detected without (**A**) and with (**B**) Spectra.

Structural assessment and preoperative functional mapping allow for more accurate predictability of patients who are eligible for gross total or supramaximal resection [46,47]. The distance between the light source and the tissue of interest, alongside its intensity, was related to tissue fluorescence [48,49,50]. Therefore, in patients eligible for fluorescence-guided GTR based on preoperative assessment, Spectra can provide increased 5-ALA fluorescence when inspecting the cavity after bulk tumour resection in both open surgical resection and tubular access cases. Figure 6 and Video S4a,b provide good examples of residual fluorescence detected with Spectra that could not be identified with the blue-light source on the microscope only.

#### 3.1.3. Satellite Foci Identification and Resection

**Figure 7:**
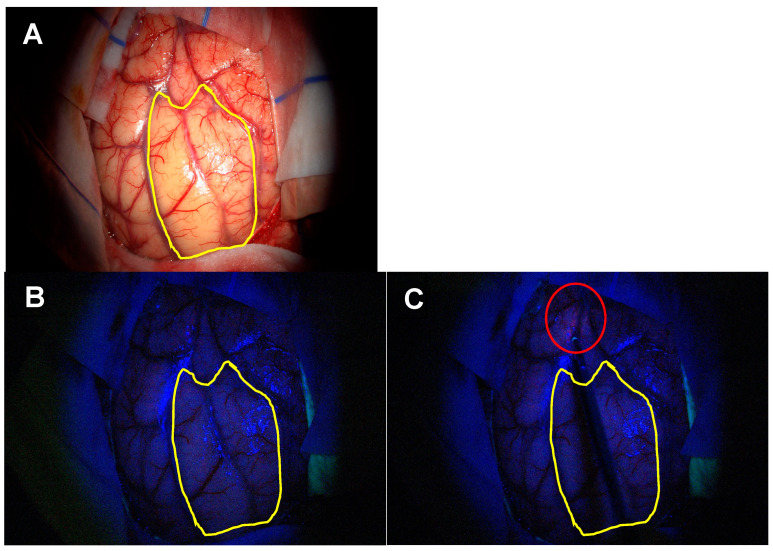
Satellite lesion identification and resection. (**A**) Tumour identification (yellow contouring) according with the neuronavigation, the anatomical landmarks and the microscopic view with evidence of bulging of the surface and effacement of the sulci. (**B**) Absence of fluorescence under the blue light of the microscope. (**C**) Satellite spot of fluorescence (red contouring) with the use of Spectra.

**Figure 8:**
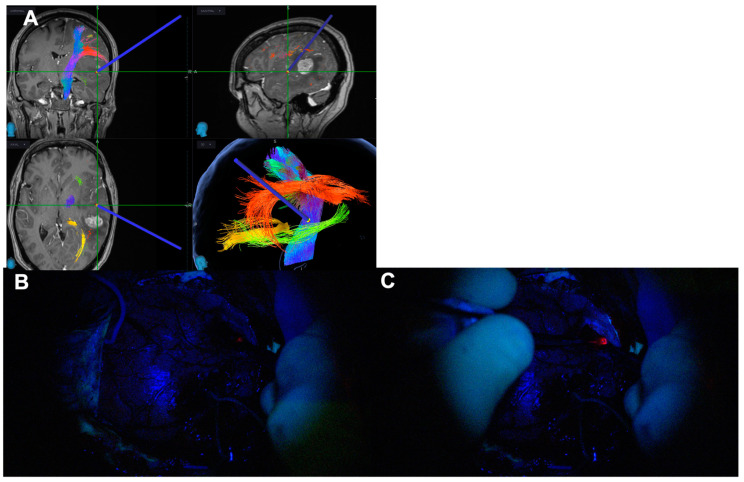
Satellite lesion identification and resection. (**A**) Satellite lesion confirmed with neuronavigation. (**B**) The nodule is not visible under the blue light of the microscope, but it becomes fluorescent with Spectra (**C**).

Although the nomenclature of multifocal and multicentric glioblastoma is still a matter of discussion, the presence of satellite foci is a common feature in high-grade gliomas [51,52]. Often, there is no continuation in the 5-ALA fluorescence between the main lesion and the satellite lesions. A focal blue-light source provides enhanced visualisation that assists, in combination with neuronavigation or intraoperative imaging adjuncts (MRI, CT or ultrasound), in the identification of small satellite lesions. Figure 7 and Figure 8 illustrate how the resection towards the satellite lesion is guided using the Spectra.

#### 3.1.4. Differentiation Between Necrosis and Recurrence in Redo Surgery

**Figure 9:**
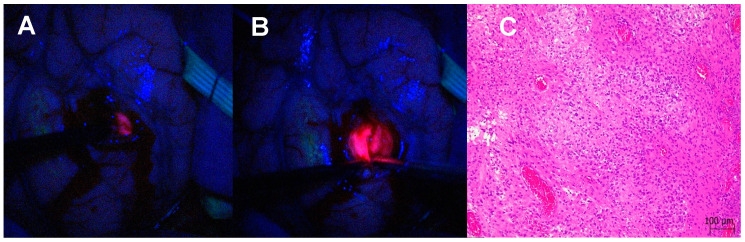
Differentiation between necrosis and recurrence in redo surgery. (**A**) Microscopic view without Spectra. (**B**) Spectra-enhanced fluorescence of the tumour tissue. (**C**) Histological confirmation of pathologic features in the tissue visualised with Spectra with the tumour core densely cellular with markedly atypical astrocytic cells, microvascular proliferation (right side) and necrosis on the left side (H&E, 10× magnification).

In redo high-grade glioma surgery, resection is usually guided by neuronavigation, anatomical landmarks, the relationship with the pre-existing cavity and microscopic intraoperative visualisation of the neoplastic tissue compared to the non-tumoural border. Fluorescence under the blue light of the microscope can be extremely helpful in differentiating the recurrent tumour from the surrounding tissues [53]. With the Spectra, we experienced a further amplification of the pathologic tissue and, consequently, a lower risk of missing the tumour during the redo resection. Figure 9 shows the amplified fluorescence appreciated with and without Spectra and the histological confirmation of the tumour tissue analysed.

#### 3.1.5. Identification of Normal Ependyma Versus Pathological Ependymal and SubepenDymal Tissue

**Figure 10:**
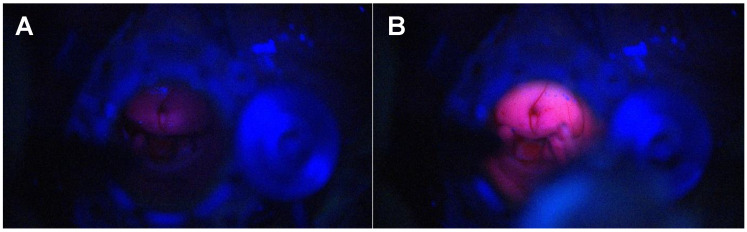
Identification of normal ependyma versus pathological ependymal and subependymal tissue. (**A**) Blue-light microscopic view of invaded neoplastic ependyma without Spectra. (**B**) Enhanced pathological ependymal fluorescence visualised with Spectra.

Ventricles represent an anatomical landmark during tumour resection. The pathological significance of ependymal fluorescence is still debated, and no clear differentiation has been proved between pathological and non-pathological fluorescence [54,55,56]. In our series, under the blue light of the microscope, the ependymal and subependymal fluorescence did not diverge significantly while, with Spectra, we could appreciate different degrees of fluorescence between the ependymal wall and the adjacent region invaded by the tumour, in keeping with the imaging (Figure 10).

### 3.2. Surgical Workflow During 5-ALA-Guided Resection

**Figure 11:**
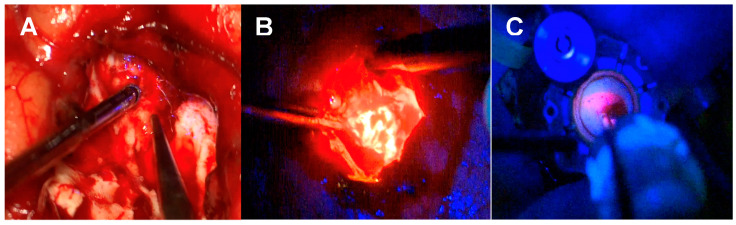
Surgical workflow during 5-ALA-guided resection with the 3 different setups used during the procedures. (**A**) Microscope, White Light/Spectra, Blue Light. (**B**) Microscope, Blue Light/Spectra, White Light. (**C**) Microscope, Blue Light/Spectra, Blue Light.

Three different setups were used during these procedures (Figure 11A–C):

1. Microscope—White Light; Spectra—Blue Light;
2. Microscope—Blue Light; Spectra—White Light;
3. Microscope—Blue Light; Spectra—Blue Light.

The first setup does not work as the external white light saturates the focally delivered blue light and therefore, no metabolic information can be obtained from selective tissue fluorescence. The other two setups work well and provide different and complementary information. When both sources deliver blue light, the intensity of fluorescence is increased, which facilitates the identification of residual tumour, particularly in areas that may be prone to false negatives due to poor illumination—collapsed cysts within a high-grade glioma, or tumour underneath non-involved brain tissue (overhang effect). When the blue light is provided by the microscope and the white light by the Spectra, an image with focal anatomical detail provided by the white light on a background of oncological information provided by the blue light is obtained. Spectra provided the ability to change between the blue- and white-light sources with a foot pedal control with no glaring on the optical image obtained in the microscope, providing a more efficient workflow during tumour resection (Video S5). The authors work with the blue-light source on the microscope and change the light source in Spectra according to the specific information they require.

### 3.3. Spatially, Functionally and Oncologically Informed Tissue Collection

**Figure 12:**
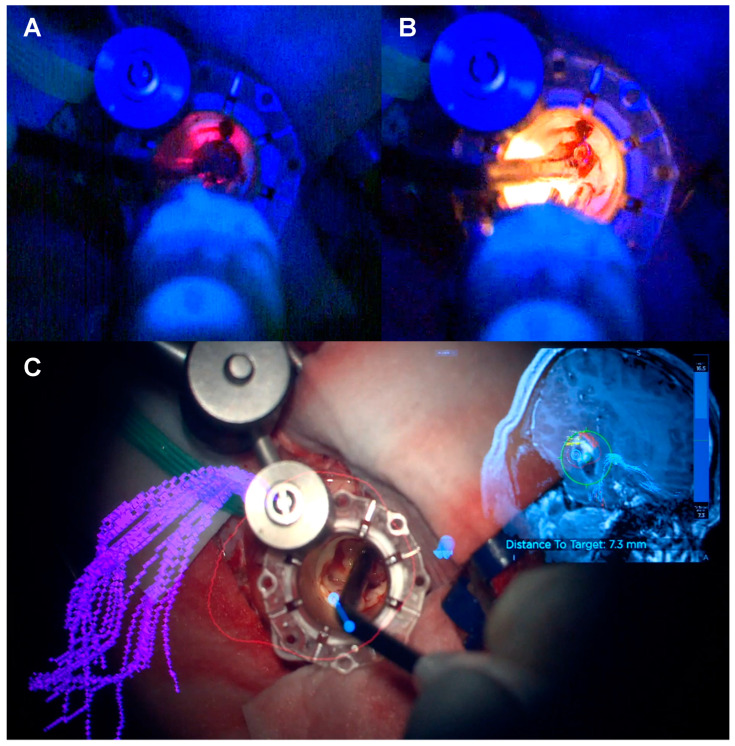
Spatial, functional and oncological informed tissue collection. The 3-in-1 approach. (**A**) Oncological information of fluorescent tumour tissue with the Spectra Blue Light. (**B**) Spatial information with better visualisation of the anatomical structures thanks to the Spectra White Light. (**C**) Spatial and functional information with the neuronavigated microscope and continuous subcortical stimulation technique.

In situ blue-light-source delivery associated with the electrification of the Nico microdebrider and navigation [42] allows for a spatially, functionally and oncologically informed tissue collection. The continuous navigation of the microscope allows tracking of the instrument position in the navigation system. There is also the possibility of navigating the Nico Myriad System itself, which provides further spatial resolution but hampers the manoeuvrability of the system given the extra weight caused by the navigation adjunct [42]. Due to continuous electrification and stimulation and using the high-frequency stimulation paradigm extensively validated in the literature, functionally informed surgery can be performed with no need for further instrumentation [38]. This is particularly useful for surgeons used to the dynamic subcortical stimulation technique as the information provided is similar and there is a sense of familiarity in the technique. Lastly, the in situ delivery of a 405 nm light in a patient with the administration of 5-ALA will provide the oncological information necessary to guide the surgical resection. This three-in-one approach is illustrated in Figure 12 and Video S6.

## 4. Discussion

**Figure 13:**
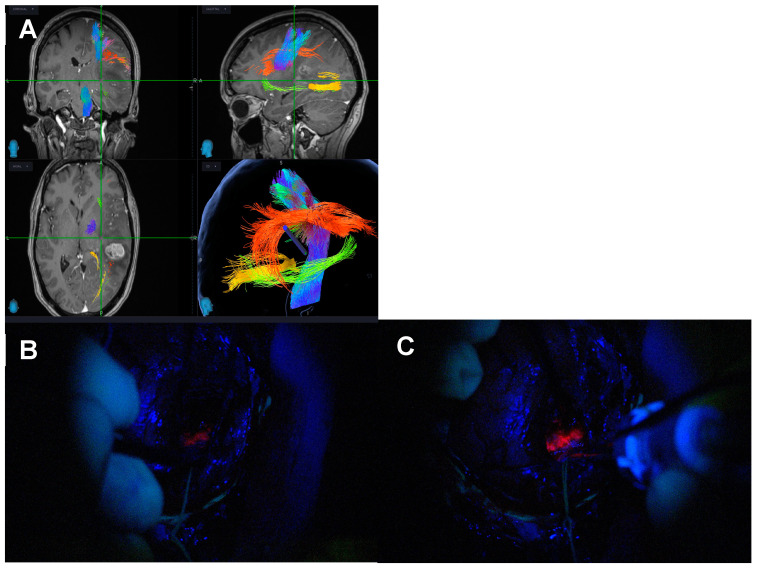
Beyond contrast enhancement. (**A**) Non-contrast-enhancement tumour confirmed in the navigation. (**B**) Visualisation of the tissue with the blue light of the microscope. (**C**) Visualisation of the same tissue with the Spectra, with enhanced fluorescence.

In situ light-source delivery provided by the Nico Myriad Spectra System© allows for enhanced tumour visualisation due to closer excitation source to the fluorophore within the tumour tissue (Figure 4, Figure 5, Figure 6, Figure 7, Figure 8, Figure 9, Figure 10, Figure 11, Figure 12 and Figure 13 and Videos S3–S6). By doing this, it promotes a spatial, functional and oncological informed framework. This multimodal approach has been advanced by integrating the Nico Myriad Spectra System© with surgical navigation (spatial), mapping techniques and the intraoperative neuromonitoring (functional) and the use of blue light and the fluorescence-guided assisted surgery (oncological informed) (Figure 12 and Video S6). This technology proved, in our experience, to be particularly useful in increasing the number of patients that can achieve GTR due to improved fluorescence visualisation of satellite foci and pathological tissue in the periphery of the surgical cavity, and in providing better tissue discrimination in redo surgery and in subventricular areas.

One of the main applications of the Spectra is its use in tubular retractor-assisted approaches (Figure 4 and Figure 5, Video S3). Visibility in minimally invasive surgery for deep-seated lesions remains challenging, and several different techniques have been experimented to overcome this obstacle, such as different shapes of retractors, different materials or the use of endoscopes [57,58,59,60,61,62,63]. The in situ light-source delivery approach extends the concept of illuminating the dark corridor of the tubular retractor to illuminate the corners of the surgical field (Figure 5 and Figure 12, and Videos S3, S5 and S6). It also minimizes the confounding effect of the presence of blood or haemostatic agents, the light angulations of the microscope or simply the hidden tissue in the corners and in the dark corridors (Figure 13 and Videos S4 and S5). The limitations in surgical visibility are considerably more noticeable when working under blue light with further additional issues related to the subjective pale fluorescence and the alternation between white and blue light [10,23,64,65]. Consequently, we applied the same principle of uncovering the hidden corner, also during the resection in the blue light setting (Figure 4, Figure 5, Figure 6 and Figure 11, Videos S3–S5). The light positioned on the tip of the instrument and the swift switch from white to blue light as soon as the foot pedal is pressed (Figure 2 and Videos S1 and S2) overcomes the illumination disruption once the microscope light switches from white to blue and vice versa.

**Figure 14:**
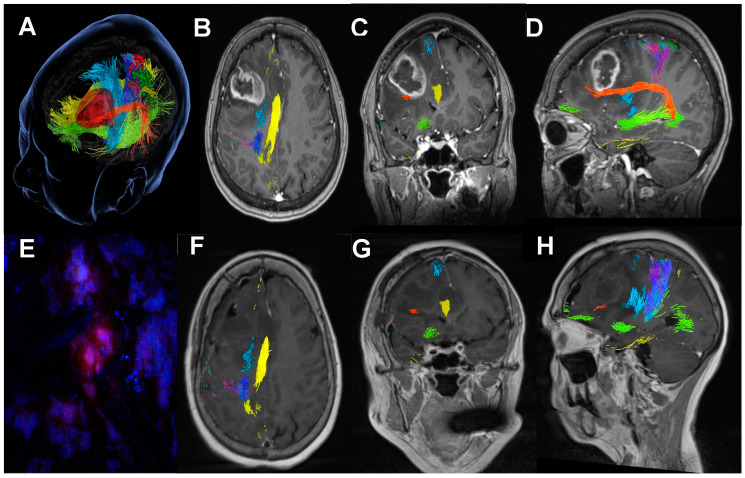
Pre- and post-operative MRIs of a patient operated with Spectra with complete supramaximal resection of a glioblastoma. (**A**) Preoperative planning with 3D reconstruction of the functional tracts. (**B**–**D**) Axial, coronal and sagittal views of the preoperative T1 contrast-enhanced MRI with the lesion and the functional tracts. (**E**) Visualisation of fluorescent residual tissue at the bottom of the resection cavity not visible without Spectra. (**F**–**H**) Axial, coronal and sagittal views of the post-operative T1 contrast-enhanced MRI with evidence of complete resection of the lesion.

Improved tissue differentiation is a crucial concept in tumour surgery facilitated by this technique that is materialised in potential to increase the EoR (Figure 14) given the better visualisation of satellite lesions within the otherwise unremarkable white matter (Figure 7 and Figure 8) and enhanced discrimination between necrosis/radionecrosis (Figure 9) and normal ependyma from tumour-infiltrated tissue (Figure 10). This has the potential to improve the quality of tissue harvested for diagnosis, which may improve molecular and genetic diagnosis and, therefore, personalised therapies either in the context of clinical trials or as second-line therapy at the time of progression in standard of care.

### Strength and Limitations

We identified some limitations and pitfalls in this technique, which we highlight as follows. Tumour or brain tissue can obliterate the light source as this is very close to the tissue resection site. If this situation goes unrecognised, a false-negative result is obtained. This can be avoided by regularly checking with the light source probe (Spectra) and continuous integration with the other techniques and the light source from the microscope. The likelihood of tumour presence should always be present during the resection and raise suspicion of unexpected negative fluorescence. Also, we have not identified a situation where we identified fluorescence with the blue light from the surgical microscope and negative fluorescence with the Spectra-derived blue source. Therefore, if this situation is encountered, suspicion of blockage of the light source should be considered. An inaccurate alignment of the Nico Myriad Spectra System© can cause misalignment between the light delivery and the side cutter of the microdebrider. This can introduce less accuracy in the tissue collection and the excitatory blue-light source will not be coincident with the tissue to be resected/collected. Also, other well-known pitfalls in 5-ALA-guided surgery—presence of blood and background intraventricular fluorescence [23]—are still valid and should be accounted for when using this technology.

The main strength of this technology is its integration with the existing surgical adjuncts already available for high-grade glioma surgery, which is a cornerstone to the spatial, functional and oncological informed surgery framework. In this context, the versatility of this system allows for navigation and electrification, providing real-time structural and functional information. According to the surgeon’s preference, this system can either be used in the dominant hand as a single multimodality instrument (useful in narrow surgical corridors such as in the MIPS approach) or in the non-dominant hand to provide spatial, functional and oncological information whilst the dominant hand performs tumour resection with a suction cannula or an ultrasonic aspirator in superficial and wide surgical exposures (Figure 11 and Figure 12, and Videos S3, S4 and S6). There is also the possibility of integration of Nico’s tissue preservation systems (TPSs) coupled to the Nico Myriad microdebrider that facilitate the biological preservation of tissue quality from surgery to the neuropathology laboratory [66]. Therefore, we interpret this technique as an adjunct to the neuro-oncology surgeon armamentarium to better address the high-grade glioma surgery and not necessarily as a replacement of other well-established techniques.

## 5. Conclusions

In situ white- and blue-light delivery maximises the information available to the surgical team. It improves visualisation during tubular retractor-assisted surgery and increases the extent of resection through further identification of fluorescent areas and decreasing the avoidable residual tumour due to lack of visualisation in hidden areas within the surgical field. It also enhances tissue differentiation, particularly in redo surgery and in periventricular areas. Altogether, this technique paves the way to a spatial (integration with surgical navigation), functional (integration with mapping techniques) and oncological (better fluorescence visualisation) informed surgery. Further data will be collected during the follow-up in order to correlate the intraoperative advantages of the technique with the long-term clinical and radiological outcomes, such as the post-operative findings in the follow-up MRIs, overall survival and progression-free survival.

## Figures and Tables

**Table 1:** Data of the patients (sex, age, tumour location, procedure performed, diagnosis and extent of resection).

	Sex	Age	Tumour Location	Procedure	Diagnosis	Extent of Resection
1	M	60	Temporal	Right fronto-temporal craniotomy	Glioblastoma, IDH-wildtype, CNS WHO grade 4	GTR, no residual fluorescence
2	M	66	Temporo-parietal	Awake left temporo-parietal craniotomy	Glioblastoma, IDH-wildtype, CNS WHO grade 4	STR due to eloquent area, residual fluorescence
3	F	56	Paracentral lobule	Left parasagittal fronto-parietal craniotomy	Metastatic non-small cell carcinoma	GTR, no residual fluorescence
4	M	39	Temporal	Redo right temporal craniotomy	Residual/recurrent Glioblastoma, IDH-wildtype, CNS WHO grade 4	GTR, no residual fluorescence
5	F	58	Temporo-parietal	Awake left fronto-temporo-parietal craniotomy for tr-MIPS	Glioblastoma, IDH-wildtype, CNS WHO grade 4	STR due to eloquent area, residual fluorescence
6	M	72	Parieto-occipital	Redo right parieto-occipital craniotomy	Marked reactive/therapy-related changes with a tiny cluster of residual metastatic adenocarcinoma	GTR, no residual fluorescence
7	M	60	Frontal	Left frontal craniotomy	Meningioma (in the context of BAP1 tumour predisposition syndrome)	GTR, no residual fluorescence
8	M	61	Temporal	Left fronto-temporal craniotomy	Glioblastoma, IDH-wildtype, CNS WHO grade 4	STR due to invasion of vascular structure, residual fluorescence
9	F	45	Temporal	Right temporal craniotomy for tr-MIPS	Subependymoma WHO grade 1	GTR, no residual fluorescence
10	F	73	Temporal	Left fronto-temporal craniotomy	Glioblastoma, IDH-wildtype, CNS WHO grade 4	GTR, no residual fluorescence
11	F	70	Temporal	Right fronto-temporal craniotomy	Glioblastoma, IDH-wildtype, CNS WHO grade 4	STR due to invasion of vascular structure, residual fluorescence
12	F	52	Temporo-parietal	Redo right temporo-parietal craniotomy	Residual/recurrent glioblastoma, IDH-wildtype, CNS WHO grade 4	GTR, no residual fluorescence
13	F	63	Parietal	Right parietal craniotomy for tr-MIPS	Glioblastoma, IDH-wildtype, CNS WHO grade 4	STR due to invasion of vascular structure, residual fluorescence
14	M	55	Temporal	Left temporo-parietal craniotomy	Glioblastoma, IDH-wildtype, CNS WHO grade 4	GTR, no residual fluorescence
15	M	35	Temporal	Right fronto-temporal craniotomy	Glioblastoma, IDH-wildtype, CNS WHO grade 4	GTR, no residual fluorescence
16	F	23	Frontal	Awake redo left frontal craniotomy	Recurrent astrocytoma, IDH-mutant, CNS WHO grade 3, transformed from lower-grade astrocytoma	STR due to eloquent area, residual fluorescence
17	M	51	Temporal	Left temporal craniotomy for tr-MIPS	Glioblastoma, IDH-wildtype, CNS WHO grade 4	STR due to eloquent area, residual fluorescence
18	F	51	Frontal	Awake right frontal craniotomy	Glioblastoma, IDH-wildtype, CNS WHO grade 4	GTR, no residual fluorescence
19	F	34	Frontal	Awake right fronto-temporo-parietal craniotomy	Oligodendroglioma, IDH-mutant and 1p/19q co-deleted, CNS WHO grade 2	STR due to eloquent area, no fluorescence
20	M	79	Frontal	Right frontal craniotomy	Glioblastoma, IDH-wildtype, CNS WHO grade 4	STR due to eloquent area, residual fluorescence
21	M	66	Parietal	Awake right fronto-temporo-parietal craniotomy	Glioblastoma, IDH-wildtype, CNS WHO grade 4	STR due to eloquent area, residual fluorescence
22	F	52	Fronto-temporo-parietal	Awake right fronto-temporo-parietal craniotomy	Glioblastoma, IDH-wildtype, CNS WHO grade 4	STR due to eloquent area, residual fluorescence
23	F	29	Frontal	Awake right frontal craniotomy	Diffuse low-grade glioma, IDH-mutant, favouring oligodendroglioma CNS WHO grade 2	GTR, no fluorescence
24	M	79	Parietal	Right parietal craniotomy	Metastatic mucinous adenocarcinoma, in keeping with primary lung origin	GTR, no residual fluorescence
25	M	35	Frontal	Awake right frontal craniotomy	Oligodendroglioma, IDH-mutant and 1p/19q-codeleted, CNS WHO grade 2	STR due to eloquent area, no fluorescence
26	M	31	Thalamic-intraventricular	Left parietal craniotomy for tr-MIPS	Low-grade glioneuronal tumour, favouring ganglioglioma, CNS WHO grade 1	STR due to eloquent area, no fluorescence
27	M	35	Temporal	Right fronto-temporal craniotomy	Glioblastoma, IDH-wildtype, CNS WHO grade 4	GTR, no residual fluorescence
28	M	59	Temporal	Right fronto-temporal craniotomy	Glioblastoma, IDH-wildtype, CNS WHO grade 4	GTR, no residual fluorescence
29	F	44	Temporal	Awake right fronto-temporal craniotomy	Glioblastoma, IDH-wildtype, CNS WHO grade 4	STR due to eloquent area, residual fluorescence
30	M	54	Temporal	Right fronto-temporal craniotomy for tr-MIPS	Glioblastoma, IDH-wildtype, CNS WHO grade 4	GTR, no residual fluorescence
31	M	56	Frontal	Left frontal craniotomy	Glioblastoma, IDH-wildtype, CNS WHO grade 4	GTR, no residual fluorescence
32	M	30	Frontal	Left frontal craniotomy for tr-MIPS	High-grade astrocytoma with piloid features	GTR, no residual fluorescence
33	F	62	Frontal	Awake left frontal craniotomy	Glioblastoma, IDH-wildtype, CNS WHO grade 4	GTR, no residual fluorescence
34	M	72	Posterior insula	Awake left parietal craniotomy for tr-MIPS	Diffuse large B-cell lymphoma (DLBCL), strongly suggestive of a non-germinal centre B-cell-like (non-GCB) subtype	STR due to eloquent area and intraop results, no fluorescence
35	F	34	Left cerebellar	Suboccipital craniotomy	Medulloblastoma	GTR, no residual fluorescence

## Data Availability

Data about the new technology can be found on the website https://niconeuro.com/our-integrated-system/ (accessed on 3 November 2024).

## Supplementary Materials

The following supporting information can be downloaded at: https://www.mdpi.com/article/10.3390/biomedicines12122748/s1; Video S1: Practical demonstration of the use of the foot pedal. Switch between white light and blue light of the Spectra under the white (a) and blue (b) light settings of the microscope; Video S2: Example of use of the Spectra Nico Myriad, in the BrainPath tubular retractor, either using the white light or the 400 nm wavelength light source from the microscope, with either white light or blue light (400 nm) at 100%; Video S3: Application of Spectra in Tubular Retractor–Minimally Invasive Parafascicular Surgery (tr-MIPS); Video S4 (a,b): Examples of visualisation of the residual fluorescence at the bottom of the surgical cavity thanks to the use of the Spectra; Video S5: Surgical workflow during 5-ALA-guided resection. Example in tr-MIPS with alternation of the use of the different settings; Video S6: Example of the 3-in-1 approach (spatial, functional and oncological informed technique).
